# “Rape Is a Big Word to Describe It”: Reframing Marital Sexual Violence in Muslim Majority Contexts—The Case of Egypt

**DOI:** 10.1177/10778012251410227

**Published:** 2026-02-02

**Authors:** Dina Magdy Taha, Nayera Mohamed Shousha

**Affiliations:** 1 Department of Sociology and Anthropology, 518164 Doha Institute for Graduate Studies, Doha, Qatar; 2 Department of Social Psychology, 518164 Doha Institute for Graduate Studies, Doha, Qatar; 3 63526 Cairo University, Cairo, Egypt

**Keywords:** marital sexual violence, marital rape, intimate partner sexual violence, Muslim-majority, Egypt

## Abstract

Based on 13 qualitative interviews with Egyptian women who experienced sexual violence by their husbands, this study examines whether “marital sexual violence” (MSV) better captures perceptions in Muslim-majority communities than terms like “marital rape” or “intimate partner sexual violence.” The analysis reveals that while many women acknowledged the violence, they refrained from labeling it as rape, reflecting ongoing debates over terminology. MSV, we argue, may offer culturally responsive framing by shifting the focus from consent (seen as a woman's duty) to harm (a man's accountability). This approach bridges gaps between everyday experiences and academic discussions, facilitating meaningful dialog across social, advocacy, and religious contexts and centering community-based and decolonial interventions.

## Introduction

The notion of marital rape as a human rights and gender-based violence crime is rooted in Western legal traditions, which historically denied the possibility of rape within marriage. Although this framework evolved over time, ultimately leading to the criminalization of forced sexual acts within marriage (Bennice & Resick, 2003), the very definition and boundaries of “marital rape” remain deeply contested across global contexts (Yllö & Torres, 2016). In the Muslim-majority world, as in other regions, these debates intersect with long-standing critiques of colonial feminism, where gendered harm was often instrumentalized to justify political and economic interventions (Abu-Lughod, 2002; Razack, 2004), sometimes obscuring or depoliticizing local gender-based violence. In Egypt, one of the largest Arab and Muslim countries, narrow interpretations of Islamic jurisprudence continue to shape how sexual violence within marriage is justified or obscured, with some voices increasingly cautioning against treating religious readings as authoritative (Abourabi & Lubaale, 2021). The absence of legal recognition for marital sexual violence (MSV) further contributes to its normalization and underreporting (Ammar, 2006). Even when the Egyptian National Council for Women proposed a law in 2013 to criminalize violence against women and domestic violence, it failed to address sexual violence within marriage (Egypt Today Staff, 2017).

Some local efforts have highlighted the importance of Islamic ethical principles in marital relationships (Gaballa, 2020) and have issued reinterpreted fatwas that condemn a husband's use of violence against his wife as sinful (Allam, 2022; Dar Al-Ifta, 2013). Despite these interventions, mainstream societal views continue to emphasize men's rights and women's duties in marital sexual relations. This dynamic is reflected in family law, where sexual acts within marriage remain uncriminalized, leaving victims to face numerous legal, emotional, and social obstacles with limited formal or informal support (Mojahed et al., 2022).

At the same time, a growing body of critical and decolonial feminist scholarship highlights how colonial and Orientalist discourses have long fixated on sexuality as a key marker of Otherness, deploying it to justify a White savior narrative (Farahani & Thapar-Björkert, 2020; Spivak, 1988) and advance self-serving civilizing missions (Ahmed, 1992). These critiques underscore the need for more nuanced and contextually grounded understandings of non-Western gendered dynamics, tensions, and negotiations. Against this backdrop, this study employs a critical feminist approach to examine the understudied phenomenon of sexual violence within marriage, often dubbed “marital rape,” in Muslim-majority contexts through the case of Egypt. At the same time, it aims to challenge reductionist narratives and foster a deeper understanding of the complexities inherent in gendered relations, including sexual and gender-based violence (SGBV).

The current study is an extension of a research project that investigates the phenomenon of sexual violence within marriage in Egypt (Shousha & Taha, 2025), which drew on interviews with 15 Egyptian women who had experienced various forms of sexual violence by their husbands. This exploratory study, the first of its kind to delve into this specific social and geographic context, examined the cultural, legal, social, and religious factors that shaped the women's perceptions and experiences of what is commonly referred to as marital rape, as well as the barriers they encountered in seeking both formal and informal support. The initial study concluded that while gendered power dynamics within marriages, families, and broader society contribute to the minimization and normalization of violence in marital sexual relations, they still have severe consequences for women's mental and physical health, reinforcing the hierarchy and cycle of violence.

Reflecting on the analysis, nevertheless, our decision to label the experiences of the respondents as “rape” proved challenging, as many were reluctant to characterize the intimate violence they endured as such. This hesitation aligns with broader academic debates; the terminology within this field is far from unanimous, with terms like forced sex, sexual assault, and intimate partner sexual violence (IPSV) often preferred (Yllö & Torres, 2016). Lynne Kwiatkowski (2016), for instance, documented that Vietnamese women who have experienced forced sex in marriage perceive it as abuse or violation, but not as rape. Other scholars continue to question the applicability of the concept of marital rape outside Western contexts, where it lacks historical and legal foundations, local acceptance, and recognition of the concept of consent (Torres, 2016). Naming these experiences as marital rape, often advocated by human rights activists, criminologists, and public health workers, can be perceived as an attack on the “sanctity of marriage” within some cultures which is exacerbated by a scarcity of qualitative research exploring how forced sex and sexual violence within marriage are culturally conceptualized outside of Western discourses and epistemes (Torres, 2016). This leads to a persistent challenge in redefining marital rape and related forms of IPSV, a fragmentation of effort in addressing violence within marriage and more importantly, a disconnect between people's everyday meanings and experiences on one hand and discussions of academics and activists on the other.

Thus, this article delves deeper into the narratives of the respondents to examine whether employing culturally responsive and narrowed-down terminology, such as MSV instead of marital rape or IPSV, might better capture the recognition, perception, and response to sexual violence within marital relationships. Furthermore, this distinction might offer a better entry point to the specific legal and social challenges and the chronic nature of sexual violence within marital relationships in global but specifically Muslim contexts.

## Literature Review

IPSV and marital rape are perceived as forms of gender-based violence that disproportionately affect women (Bennice & Resick, 2003; Bergen & Barnhill, 2006; Crichton et al., 2010; Moawad et al., 2021; Yilmaz, 2015; Yllö & Torres, 2016; Shah et al., 2010), which involve nonconsensual or unwanted sexual acts (Banerjee & Rao, 2022; Dubey & Verma, 2018). These acts can include physical (e.g., slapping and beating) and nonphysical coercion (e.g., insults, blackmail, constant humiliation, intimidation, and threats of harm or divorce; Bagwell-Gray et al., 2015).

Feminist research consistently argues that MSV is a form of social control and maintaining dominance over women (Finkelhor & Yllo, 1985; Johnson, 1995). This viewpoint aligns with the common stereotype of women as gentle, obedient, and nurturing, and contrasts sharply with the stereotype of men as rough, virile, and aggressive (Johansson-Nogués, 2013; Oh & Kutufam, 2014; Shousha, 2020). Yet, in many cultures, including Muslim-majority societies, however, customs and practices decenter the principle of consent in marital sexual relations (Mojahed et al., 2022; Yllö & Torres, 2016), leading to different parameters and interpretations of the act.

In the Egyptian context, there are limited studies that explore the prevalence of IPSV. Yaya et al. (2021) used data collected from the Egypt Demographic and Health Survey, which pointed out that 29.4% of ever-married Egyptian women reported experiencing some form of violence, with 26.7% facing physical violence, 17.8% emotional violence, and 4.6% sexual violence. Sexual violence, though the least prevalent, remains significant, with 4.5% of women reporting being physically forced into intercourse and 3.1% into other sexual acts. Other studies showed that factors such as women's age (25–29), Islamic beliefs, and husbands’ education were significantly associated with IPV (El-Zanaty et al., 1996). Cultural attitudes also play a key role, with many women justifying abuse due to infidelity, disobedience, or neglecting household duties (Boy & Kulczycki, 2008). The justification of violence, particularly physical abuse for refusing sex, was notably high in Egypt, with 70% of women endorsing it (El-Zanaty et al., 1996). This explains why, despite results showing that 30% of married women reported experiencing some form of spousal violence, only 4% reported sexual violence, emphasizing potentially the underreporting in the marital context (El-Zanaty et al., 1996). Compounding the issue, all forms of IPSV remain highly underreported due to societal stigma, cultural validation of spousal rights, and outdated legal frameworks (Martin et al., 2007; Yaya et al., 2021). These findings suggest a need for further and more up-to-date research on the cultural and gendered beliefs surrounding IPV and provide a safe environment for women to disclose their abusive experiences, especially sexual abuse by husbands.

Academic debates highlight the distinctions and overlap between marital rape and IPSV. However, the distinction between MSV and these two forms of abuse is less clearly defined. Marital rape refers specifically to nonconsensual sexual intercourse or penetration within marriage, obtained by force, using any form of threat, or when the wife is unable to consent (Bergen & Barnhill, 2006; Martin et al., 2007). Feminist and social constructionist theories emphasize that marital rape is rooted in the power dynamics of the patriarchal family, perpetuated through concepts like “wifely duty,” “license to rape,” and “patriarchal terrorism” (Finkelhor & Yllo, 1985; Johnson, 1995; Martin, Taft & Resick, 2007).

IPSV, which often combines both marital and nonmarital relationships, covers a wider spectrum of nonconsensual sexual acts, including emotional manipulation and coercion, with both physical and nonphysical forms of violence (Basile, 2002; Logan et al., 2015). MSV, however, is not a term commonly used in the literature, with only limited exceptions such as Hadi (2000), Chattopadhyay (2019), and McDougal et al. (2021), and even these studies offer little in the way of a clear conceptual definition. Nevertheless, evidence suggests notable distinctions between marital and nonmarital intimate partner sexual violence, particularly in terms of severity and consequences. Marital IPSV often involves more severe physical harm, including the use of weapons, rape, and other forms of invasive sexual assault, compared to nonmarital IPV, where forcible fondling is more common (Browne, 1993; Coker et al., 2000; Krienert & Walsh, 2018). Victims of marital IPSV are also more likely to suffer long-term emotional and psychological damage, such as posttraumatic stress disorder, anxiety, and depression, as well as increased risks for repeated victimization (Bennice & Resick, 2003; McFarlane et al., 2005). These victims may experience deeper feelings of betrayal due to the violation of marital trust, which exacerbates their trauma (Bergen & Barnhill, 2006). For instance, research shows that victims of MSV are more likely to experience severe forms of abuse, including rape and injury, compared to non-MSV, where forcible fondling is more common (Coker et al., 2000).

We posit, thus, that MSV, is a distinct term encompassing additional challenges for women victimized by their husbands, including legal, financial, familial, and social restrictions, which create further barriers to leaving an abusive marriage or even reporting the abuse (Apatinga & Tenkorang, 2025; Rohn, 2021). MSV is particularly useful in cultural contexts (such as Egypt) where the concept of marital rape is controversial or debated which might further silence victims. Additionally, marriage is often the only socially and religiously accepted intimate relationship in such contexts. Thus, exploring this research gap is crucial for obtaining a comprehensive understanding of the challenges faced by victims and the role of cultural perceptions in addressing this stigmatized issue.

## Background: Egyptian Legal and Social Context

Egypt's family (personal status) law emerged as a hybrid settlement in the late 19th–early 20th centuries, when reformers translated Hanafi rules into numbered statutes and reorganized courts along European lines, producing “Islamic content in a civil-law form” (Cuno, 2015). Ayoub (2022) argues this was part of state-building: civil law ended legal pluralism by absorbing and managing *sharīʿa* within a centralized judiciary rather than leaving it to parallel courts. As Abu-Odeh (2004a, 2004b) details, 20th-century reforms, shaped by French legal models and British colonial governance, advanced mainly through procedural and institutional shifts while identity politics constrained substantive gender-egalitarian change.

From its inception, the Egyptian feminist movement identified personal status law as central to women's subordination. Early feminists such as Huda Sha‘rawi and the Egyptian Feminist Union pressed for reforms to polygamy, divorce, and obedience laws, while still operating within a framework of “equality in difference” (Abu-Odeh, 2004a; Badran, 1995; Karam, 1998). Yet, as Abu-Odeh (2004) notes, family law remained Islamic in substance even as other codes were secularized, making it the symbolic anchor of cultural authenticity. This “identity debate,” whether laws should be Islamic or secular, trapped feminist reform. Limited gains, such as the abolition of *bayt al-ṭāʿa*, a former legal “house of obedience's mechanism” compelling wives to return to their husbands, in 1967, were possible, but deeper reforms were blocked or reversed when they threatened the religious legitimacy of the state.

Al-Ali (2004) situates Egyptian feminism within these broader political transformations. Women's rights were repeatedly subordinated to nationalist projects, then co-opted by “state feminism” under Nasser, Sadat, and Mubarak. While state feminism expanded women's education and employment, it left patriarchal family law intact, presenting first ladies as symbolic champions of women's empowerment. At the same time, Islamist and nationalist movements reasserted conservative gender norms, framing feminism as Western, and reinforcing the family as a cultural battleground. Contemporary feminist organizations remain resilient yet fragmented, navigating authoritarian repression, conservative counter-discourses, and donor-driven NGO agendas while struggling for legitimacy. Family law has proven especially resistant, precisely because it lies at the intersection of cultural authenticity, religious authority, and political legitimacy.

Sexual violence further illustrates these dynamics. Reda El-Danbouki of the Women's Center for Guidance and Legal Awareness notes that because marital rape is not criminalized, lawyers typically pursue divorce under *ṭalāq li-l-ḍarar* (divorce for harm). Judges alone determine the extent of harm, cases can take years, and representatives often invoke alternative claims such as impotence, abuse, abandonment, or failure of financial support (Anees, 2024). Even then, divorce is not guaranteed. Article 60 of the Penal Code further permits acquittal of husbands for violence if deemed in line with sharīʿa, reinforcing “legitimate chastisement” (Ali, n.d.). Social stigma also discourages reporting sexual abuse (Abdelaal, 2021).

The Arabic term for rape, “*ightisab*” (usurpation), implies illegitimate acquisition. Under Article 267 of the Egyptian Penal Code, *ightisab* is defined as sexual intercourse with a woman without her consent and is punishable by death or life imprisonment. The death penalty is mandated if the victim is under eighteen years old, if the perpetrator is a legal guardian or holds authority over the victim, is employed as her servant, or if multiple offenders participated in the crime. Article 268 of the Egyptian Penal Code further provides that assault (*hatk ʿird*) committed by force or threat, as well as attempted assault, is prescribed aggravated imprisonment. When the victim is under eighteen or when the perpetrator is among those identified in Article 267, the penalty shall be aggravated imprisonment for not less than 7 years. If both circumstances apply, the sentence shall be life imprisonment (Said, 2020).

Egyptian criminal law does define *ightiṣāb* (rape) as nonconsensual intercourse, punishable by life imprisonment or death under aggravating circumstances, but traditional jurisprudence does not recognize “marital rape,” since marriage is framed as granting husbands legitimate sexual rights in exchange for mahr and financial responsibilities (Ali, 2016; Masud, 2022; Yilmaz, 2015). Within this framework, refusal of sexual relations is often deemed sinful (Masud, 2022), perpetuating the belief that rape cannot occur within marriage (Masud, 2022) and leaving it legally and culturally tolerated (Abourabi & Lubaale, 2021; Mojahed et al., 2022).

At the same time, central Islamic concepts continue to structure law and culture. In *sharīʿa*, mahr secures a wife's financial rights, *ḍarar* (harm) provides grounds for divorce, and *mawadda* (affection) and *raḥma* (mercy) embody the ethical ideals of marriage. Egyptian law maintains these principles: mahr remains a contractual requirement (often split into advance and deferred portions), *ḍarar* is codified as a divorce ground, and legislators frame personal status law in terms of *mawadda* and *raḥma*. Yet in practice, dominant discourses often distort them; mahr is misread as a price for sexual access, *ḍarar* imposes a heavy evidentiary burden on women, and *mawadda* and *raḥma* remain symbolic, failing to secure women's bodily autonomy (Barlas, 2019).

Egyptian feminists such as Nawal El Saadawi (1982) and Islamic feminist scholars like Kecia Ali (2016), Asma Barlas (2019), and Amina Wadud (1999) have therefore challenged patriarchal interpretations by highlighting religious resources that affirm women's sexual agency and emphasize reciprocity, justice, and compassion in marriage. These reinterpretations provide important intellectual foundations for feminist movements seeking to contest restrictive personal status laws and advocate for more egalitarian family law reforms. Thus, there are several cultural, historical, religious, and legal forces that intersect to construct a gendered social order in which women and men internalize expectations of responsibility, right, duty, obedience, and self-sacrifice, which Collins (1990) has notably referred to as the “matrix of domination.” This intersectional configuration shapes how women evaluate coercion and sexual violence within marriage, not only as an act between two individuals but as one embedded within moral, familial, societal, and religious obligations.

## Research Method

### Participants and Data Collection

A qualitative approach was employed to understand the women's perspective and definition of sexual violence within marriage *on their own terms*. Primary data was collected through online advertisements through the authors’ social network to recruit the participants. Online in-depth semistructured interviews were conducted via platforms such as Google Meet, Facebook Messenger, and Zoom, lasting 45–60 min each. Conducting the interviews online was suitable and necessary for two main reasons: first, to protect women's privacy and safety. Although video calls were initially offered, most women preferred voice-only interviews to maintain confidentiality. Second, women were geographically dispersed across different cities of Egypt; thus, the online format minimized the logistical and geographical challenges.

Open-ended questions were asked to avoid leading the women toward specific responses (see appendix). Due to the sensitive scope of the research issue, a purposive snowball sampling technique was adopted. This method involves selecting participants who meet specific criteria, which were women who self-identified as being in a sexually abusive marital relationship for at least one year, then asking them to recommend others who might also be suitable for the study. Out of an initial group of 15 educated Egyptian women who participated in the study, 13 agreed to a follow-up exploring the terminology of marital rape and whether they defined their experiences as such (Table 1). To ensure ethical conduct, their rights as participants were explained before each interview, including assurances of anonymity, the use of responses only for scientific purposes, and their rights to withdraw at any point without repercussions. Additionally, mental health resources were available for participants who displayed distress or requested support. Finally, informed consent was obtained verbally or in writing (using pseudonyms) before each interview.

**Table 1.** Respondents’ Demographics.

Women	Age	Education level	Occupation	Class	No. of marriages	Marriage years	No. of children	Marital status
Amira	24	Master	Housewife	Middle	1	2	1	Married
Rabab	42	Undergraduate	Public sector	Middle	1	22	3	Married
Yasmeen	47	Undergraduate	Public sector	Middle	5	5	5	Divorced
Samya	39	Undergraduate	Housewife	Middle	1	15	2	Married
Gehan	40	Undergraduate	Housewife	Middle	1	10	2	Divorced
Amina	44	Undergraduate	Housewife	Middle	1	13	4	Divorced
Karima	42	Undergraduate	Housewife	Middle	1	16	2	Divorced
Noura	39	Master	Public sector	Upper middle	1	15	3	Married
Doaa	45	Undergraduate	Private sector	Middle	2	20	3	Married
Aya	38	High school	Private	Middle	1	17	3	Divorced
Marwa	32	Master	Private	Upper	1	2	0	Married
Nourhan	37	PhD	Private	Upper middle	2	2	0	Divorced
Samira	42	Undergraduate	Housewife	Middle	1	22	2	Married

This table demonstrates that 12 women have attained higher education, and only one has a high school degree. Women's ages ranged between 24 and 47 years (seven out of 13). They are still married. The duration of their marriages varies between 2 and 22 years (10 out of 13) are classified as middle class, and (two out of 13) do not have children.

### Data Analysis and Positionality

All interviews were conducted and transcribed verbatim in Arabic, with original quotes retained during analysis to avoid losing cultural nuance. Key expressions were transliterated, such as *Rifqan bil-Quwarir* (“be gentle with the glass vessels”) and *Al-Malāʾika hatulaʿannī* (“the angels will curse the disobedient wife”), to preserve their cultural and religious significance. This approach ensured authenticity and empowered participants to voice their perspectives in their own terms. Using narrative analysis, we explored how participants construct and convey their experiences, capturing the complexities and nuances of their stories. In so doing, we have also incorporated affective witnessing (Richardson & Schankweiler, 2020, 2020) to engage the researchers with participants’ experiences and narratives not only intellectually but also emotionally and empathetically. Richardson and Schankweiler (2020, p. 237) explain how Affective witnessing “is a relational account” that centers “encounter, embodiment, affect and intensities of experience.” It, thus, requires the researcher to be fully present and responsive to the emotional aspects of participants’ narratives, allowing the emotions and affective dimensions of their stories to inform the interpretation and understanding of the data. In our analysis of the respondents’ narratives, we paid close attention to the affective aspects of their stories, such as tone, expression, and emotional intensity, as well as their effect on us as researchers, to gain a richer, more holistic understanding of participants’ experiences. Incorporating effect in methodologies is particularly powerful in qualitative research on sensitive topics such as MSV because it highlights the intersubjectivity of the emotional and empathetic engagement that influences how narratives and accounts are experienced, interpreted, and understood by both the respondents and the researchers (Knudsen & Stage, 2015).

In our analysis, we reflected, as researchers and as Egyptian Muslim women subjected to varying degrees of gender-based violence, on our emotional engagement and reflexivity in understanding and interpreting the experiences of others. This involves actively listening and responding to women's stories and experiences with empathy and sensitivity instead of preconceived ideologies or theories. This also helped us understand how our own emotions and perspectives influence our interactions with participants and the interpretation of data, emphasizing the relational aspect of research, where the researcher and participants cocreate knowledge through their interactions. The emotional connection between the researcher and participants can lead to deeper insights and a more nuanced understanding of the participants’ experiences.

## Analysis

During the interviews, four out of 13 perceived violence within intimate marital relationships as outright rape; the others refused to label it as such. Instead, they preferred terms such as coercion, violence, and blackmail, viewing it as a form of transgression within the bounds of marital rights. For instance, when asked explicitly whether they would consider their experiences as rape, Rabab disagreed by saying:

I don't like to describe it as rape; it could be coercion or compulsion. I am a woman who fears God, even if I didn't want it [to have intercourse] with my husband, I would go back and say it's his right given by God, and I must give it to him [the husband].

Her use of “coercion and compulsion” rather than “rape” indicates a struggle to find language that accurately describes her pain and indignation yet conforms to societal norms that do not acknowledge that rape can happen within marriage, causing more chronic consequences. More interestingly, she perceived sex, unpleasant as it may be, as something *she gives*, in the sense that she owes it to him, not as something *taken away* from her, highlighting the complex nature of agency, consent, and coercion in intimate relations and the marginality of the notion of consent in such dynamics. Karima's response also reflects the complex emotional response and tension as she tries to search for a language to capture her feelings: “*It's not rape; it's humiliation and degradation, but I can't call it rape because it wasn't done by a stranger. After all, he is my husband.”*

To her, the term “rape” is associated with illegitimacy and an external predator, making it incompatible in her view with the concept of marriage, where there is presumed legitimacy and kinship. The terms “humiliation” and “degradation” allow her to acknowledge her discomfort and sense of violation while still adhering to her religious duties, highlighting again the complex interplay between individual agency, marital dynamics, religion, and social structures. Noura, on the other hand, captures a subtle nuance and internal conflict where she admits going back and forth between labeling it as rape or not:

During the act, I think of it as rape, but when I thought about it alone afterward, it doesn't count as rape because it is the right of the person we call the rapist, but the problem is in how he took it. Rape is a forbidden act against the woman's will in the absence of marriage. In religious framing, a woman is not supposed to refuse her husband unless she has an excuse or is not doing it intentionally to upset him. Generally, we can call it coercion, compulsion, or violence during the relationship.

Her initial internal labeling of the experience as “rape” during the act indicates a profound emotional response that is associated with violation and nonconsent. However, her reconsideration of the term afterward also reflects a complex interplay of emotions deeply shaped by cultural and religious contexts that dictate marital roles and rights. This shift highlights a conflict between her immediate emotional experience and the cognitive reframing influenced by societal structure.

Notice that, with very few exceptions, the notion of consent was not central to the discussion, even among those who labeled it as rape. Instead, the respondents used language such as “submission,” “punishment,” “deprivation of personal freedom,” “force,” and “violence.” Many felt more confident and validated in characterizing these acts as “violence” rather than rape. This shift in terminology emphasizes man's actions and his religious responsibilities, rather than focusing on the woman's consent and her religious duties. For instance, Aya explained,

No, of course, it is violence, and he has no right to do that. They always resort to religion to tell women that if they refuse their husbands, God will be angry with them. But what about the man who beats his wife? Doesn't God get angry with him?

This perspective highlights the need to view the acts within the broader context of moral and ethical considerations, not just legal, human, or even marital rights. Labeling such acts as MSV instead of marital rape plays a pivotal role in shifting the focus from *whether* it should happen to *how* it happens. This distinction resonated better with the respondents who were reluctant to label their experiences as rape.

Furthermore, some women indicated that some occasional compromises to satisfy a husband's sexual needs are acceptable within marriage, even if they “do not feel like it,” especially if it is not a frequent thing. Rabab, for instance, explained that it is the wife's marital duty, as part of the philosophy of marriage in Islam, to protect her husband from sexual temptations (*te’iffoh)*: “*I am a woman who fears God, even if I don't want to have sex with my husband, I still say it's God's right and I must give it to him to shield him sexually.”* These expressions reveal how women's identities as “good wives,” or “pious women,” are socially produced through long-standing cultural, religious, and familial discourses. As a result, many respondents saw endurance, not refusal, as the morally appropriate response, reflecting how norms shape women's sense of value and citizenship within the family.

On the other hand, Nourhan, who was clear on labeling her experience as marital rape, underscores the complex nature of “consent” within a marital relationship:

It's marital rape because being married doesn't mean he owns me or can treat me like I'm nothing. He needs to respect my refusal and ask why I'm not able to be intimate with him when he wants me, instead of just focusing on taking me by force. […] We both need to agree, but even if the woman isn’t in the mood, a smart man can make her want it. If the relationship is healthy, the woman will engage willingly.

The quote reveals the nuanced and often ambiguous interpretation of consent in marriage. It suggests that within a marital context, the dynamics of consent are fluid and influenced by the deeper, ongoing relational contract, often referred to as *mawada (loving care or devotion)* and *ishra* (companionship), which can imbue certain expectations on both partners. This ongoing relationship might create an environment where a lack of initial enthusiasm or outright refusal can be viewed as a temporary state that can be ethically overcome through persuasion or affectionate engagement. That is, marital privilege complicates the understanding of consent, where the refusal of sex is not seen as a definitive denial of consent but rather as a negotiable part of the marital dynamic. This can lead to a situation where initial reluctance or nonconsent is perceived as something that can be changed through persuasion or coaxing, thus convincing the woman to be more accepting of initially unwanted advances. The fluidity in the interpretation of consent within marriage often leads to a higher tolerance for unwanted sexual advances. Nourhan used the phrase “*If the relationship is healthy, the woman will participate willingly,”* which introduces an important caveat; consent is not just about the one-time agreement but also an ongoing healthy, and mutual respect within the relationship. Others, such as Gehan, agreed that “*A relationship is supposed to be built on mawada and rahmah. Mutual Love and respect should be in the way they treat each other.”*

Furthermore, many of the women were neither explicit nor implicit about their dissatisfaction, let alone consent, or the level or nature of the violence they are experiencing, especially due to the chronic nature of this form of violence and the respondents’ knowledge of the repercussions of refusal. When asked to describe their reaction when their husband forces them to engage in intimate relations, many explained that they simply do not react, resist, or object for several reasons, such as the difference in physical strength; perceiving it as a religious duty or fearing that they are being sinful; anticipating physical or emotional abuse; and wanting to get it over with quickly. This adds more layers of ambiguity and complexity to MSV than other forms of intimate violence.

## Discussion

Living within a violent relationship has severe and chronic mental, social, and physical implications for women's well-being (Crichton et al., 2010). Our analysis reveals how emotional manipulation, coercion, and physical force often intertwine, resulting in profound emotional turmoil, psychological damage, and loss of self-worth. Many respondents rationalized enduring MSV as a lesser evil compared to the severe social consequences of leaving a marriage, such as homelessness, stigma, and public shame (*fedeeha*), or harm to their family and children. For some, it becomes part of the transactional nature of marriage, where women internalize tolerance for or downplay MSV to preserve family stability and social standing (Yllö & Torres, 2016). These dynamic underscores the need for culturally responsive language and interventions that acknowledge the health consequences of MSV without stigmatizing the victims, as the societal pressures on women to uphold family preservation exacerbate their suffering.

The narratives with all their emotional charge, contradictions, negotiations, and tensions provide a profound insight into how the women perceive violence in their marital intimate relations. Despite acknowledging the violence, they endure; most respondents do not classify these experiences as rape. Our analysis sought to dissect these perceptions, exploring the overlap but also distinctions between IPSV, marital rape, and MSV, and argued for the significance of these distinctions in legal, social, and cognitive contexts, as shown in Table 2.

**Table 2.** Definition and Key Differences Between Marital Sexual Violence (MSV), Intimate Partner Sexual Violence (IPSV), and Marital Rape.

Term	Definition	Key difference
IPSV	Any nonconsensual sexual act by a current or former intimate partner, regardless of marital status.	The broadest term encompasses violence in married and nonmarital relationships.
Marital rape	Nonconsensual sexual intercourse or penetration by a husband.	More specific than IPSV, it applies only to married couples. It characterizes and prejudges the act as criminal, centering consent (woman's burden and the lack of cultural relevance) instead of violence.
MSV	Any nonconsensual and unwanted sexual activity. It might be physical or nonphysical coercion, including within a marriage.	Similar to marital rape but may encompass a wide range of nonsexual coercive behaviors with social and religious aspects (e.g., blackmail, the threat of divorce or marrying another woman, preventing from financial resources), also culturally and religiously suits Egyptian society.

As noted in Table 2, rather than imposing the term “rape,” the concept of MSV, which is more descriptive and less value-laden, offers a framework that respects women's perspectives without forcing external or stigmatizing interpretations. When women do not perceive their experiences as rape, labeling them as such risks dismissing their self-understanding and imposing hegemonic epistemologies on their lived realities. In marital contexts, where husbands are often presumed to have automatic consent both legally and culturally, the issue of female consent becomes trivialized, complicating the recognition of criminality (Abdelaal, 2021; Masud, 2022; Yllö & Torres, 2016). MSV, we posit, acknowledges the harm inflicted without mandating a label that might conflict with personal or cultural context, validating women's experiences while addressing their complex position. MSV, as summarized in Table 2, diverges from both IPSV and marital rape by considering the chronic nature of violence, the malleability of the relationship and the distinct religious and legal frameworks, and convictions, that govern marriage in Muslim-majority contexts. Unlike IPSV, which encompasses violence in both marital and nonmarital relationships, MSV is specific to marriage, reflecting the legal and cultural expectations that grant husbands sexual privileges in exchange for financial and social responsibilities, including the provision of *mahr* (a form of dowry, in many cases symbolic, given to the woman; Ali, 2016; Masud, 2022; Yilmaz, 2015). These dynamics (*mahr, mowada*) complicate the recognition of nonconsensual sex as rape, as marital contracts are often viewed as reciprocal agreements where ongoing consent is presumed.

On the other hand, we consider here why referring to it as MSV rather than marital rape or IPSV might be more impactful, particularly within a Muslim context where the concept of *darar* (harm), resulting from violence, is emphasized, and where rape is traditionally understood as taking something that does not belong to you, thus not applicable within both the legal contract (represented in *mahr*) and relational contract (represented in notions such as *mawada* and the flexible room for persuasion).

Our findings thus underscore that MSV must be read as a reading produced at the intersection of cultural expectations, historical gender hierarchies, religious interpretations of marital rights, and legal logics and limitations. Women's reluctance to label their experiences as rape can be understood within a framework that considers socialization processes, which emphasize their roles and responsibilities as wives, along with fear of social repercussions and societal norms that stigmatize discussions of violence, particularly sexual violence, within marriage (Abdelaal, 2021). The absence of explicit legal criminalization of sexual violence within marriage in Egyptian law further entrenches societal acceptance (Ammar, 2006). These dynamics contribute to the isolation of women and the perpetual normalization of MSV in Egypt. That said, imposing terms like “rape,” that may not resonate with women's experiences, introduces additional barriers for those navigating patriarchal structures, as they engage in what Kandiyoti (1988) describes as “bargaining with patriarchy,” where women employ diverse strategies to cope with SGBV within these systems.

By centering the chronicity, emotional complexity and legal opacity of sexual violence within marriage, MSV offers a more nuanced framework than marital rape and IPSV, which often focus on consent and criminality in ways that may not resonate with the women's lived realities. MSV, furthermore, empowers women by creating a language that is accessible and culturally responsive, facilitating community-rooted solutions alongside activist and legal interventions. Many men may not perceive their actions as rape because they view them as part of their marital rights, a belief reinforced by patriarchal norms, but many might resonate with the notions of *darar*/violence. As one respondent noted above, however, the issue lies “not in *what* was taken, but in *how* it was taken.” This perspective challenges the centrality of consent in legal frameworks and its fluidity within the marital contexts, and underscores the need for language that captures the emotional and ethical dimensions of marital violence.

Another reason why a clear distinction between IPSV and MSV is essential is to address the specific legal and social challenges of sexual violence in marital relationships in Muslim contexts. For instance, Egypt's legal system does not criminalize sexual violence within marriage, focusing instead on physical abuse (Egypt Today Staff, 2017; Radwan, 2021). Referring to these acts as MSV allows for a deeper understanding of women's experiences and paves the way for more inclusive and decolonial approaches that engage not just legal or feminist advocacy groups but also religious, familial, and community entities. By framing these actions as MSV, the focus shifts from *intent* to *consequences*, harm and violence, thereby creating space for community-based solutions and culturally responsive sex education.

The frequent use of terms like “coercion,” “compulsion,” or “violence” by respondents highlights the need for terminology that aligns with their gendered identity and moral agency (Mahmood, 2005). Mahmood argued that women's agency in patriarchal contexts is often misunderstood within Western feminist frameworks, which tend to equate agency with resistance or autonomy. However, in contexts like Egypt, moral agency is shaped not through opposition to religious or cultural norms, but through the ethical cultivation of self within those frameworks. In this sense, women's adherence to religious and cultural norms, even when they appear submissive, can be understood as forms of moral agency where piety, duty, and personal virtue play central roles. When respondents use terms such as “coercion” and “violence” instead of “rape,” they are not necessarily negating the harm they experience. Rather, they are navigating the complex interplay between their moral and religious obligations as wives, and their lived experiences of harm. The language they choose reflects a conscious engagement with their moral subject position within the larger religious and cultural matrix.

By framing these acts as MSV, the focus shifts from *consent* to *harm*, allowing for a more comprehensive understanding that bridges the gap between intention and impact. This nuanced approach underscores the importance of ongoing dialogue about the ethics of consent and mutual responsibility in marriage. It challenges simplistic consent frameworks and calls for a deeper understanding of marital dynamics, where consent is continually negotiated. This discussion is crucial for addressing the gray areas of consent (Abourabi & Lubaale, 2021; Mojahed et al., 2022), especially in marital relationships where sexual violence is intertwined with mutual respect, familial obligations, or even punishment and revenge. Framing MSV as a harm rather than a consent issue also shifts the focus from justifying spousal abuse to scrutinizing the husband's actions under the principle of *darar* (harm), which is aligned with Islamic jurisprudence. This culturally responsive framing (un)silences women and enables them to express their experiences without feeling they are violating religious principles or cultural norms. It creates a pathway for discussing MSV within the community while validating survivors’ lived realities and choices.

## Conclusion

Defining marital rape is complex and depends on various legal, historical, and cultural factors (Yllö & Torres, 2016). In many contexts, including Muslim-majority contexts, societal dynamics do not center the principle of consent in marital sexual relations (Mojahed et al., 2022; Yllö & Torres, 2016). While we initially considered terms like marital rape or IPSV in exploring our respondents’ experiences, we ultimately chose MSV to better capture the narratives of the women interviewed in this study. These narratives reveal that although many women recognize the violence they experience, they often refrain from classifying it as rape.

We argue for using the term MSV, especially in Muslim contexts where the concept of *darar* (harm) is central, and where the traditional understanding of rape, as taking something that does not belong to one, may not apply easily within marriage contract, and where consent is more complex. Terms like “marital rape” can, thus, be seen as foreign, neocolonial or confrontational within local communities, sometimes leading to resistance, downplaying, or dismissal as part of a “Western hegemonic and morally corrupting feminist agenda” which may hinder constructive dialogue around the issue and defuse advocacy initiatives. In contrast, MSV provides a more enabling language that encourages community engagement and dialogue beyond legal and advocacy interventions. It acknowledges the unique legal and social aspects of sexual violence within marriage compared to other forms of IPSV and offers a clearer framework for holding men accountable while validating women's experiences.

By focusing on harm and impact rather than criminality and consent, MSV facilitates discussions that align with social and religious contexts, making the issue more accessible and less controversial, and aligning more comfortably with social and religious entry points without compromising on feminist egalitarian worldviews. Focusing on violence and harm also makes it more difficult to dismiss. Communities resembling the Egyptian context are more likely to engage in meaningful conversations about harm and violence than about marital rape, which carries heavy connotations of criminality and moral failure. Eventually, examining MSV in the Egyptian context highlights the intersection between Egyptian law regarding domestic violence, Sharia, and customary norms, which often reveals tensions between modern legal provisions and deep-rooted social practices. Understanding the language used to describe their experiences and the frameworks through which harm is recognized becomes crucial for understanding how family dynamics, gender relations are both constructed and contested in contemporary Egypt.

A conceptual distinction between MSV, IPSV, and marital rape, thus, acknowledges the unique legal, mental, and health implications of sexual violence in Muslim-majority marital contexts. Legal frameworks that emphasize MSV can shift the focus to harm and violation within marriage, sidestepping the contentious concept of rape. MSV provides a culturally resonant language, redirecting attention to the husband's harmful actions rather than the woman's consent, and upholding the principle that no one has the right to cause harm to anyone, from any gender, even within marriage.

## Limitations and Future Research

This exploratory study, based on a limited sample of Egyptian women, may not fully represent the experiences of women across different socioeconomic backgrounds. While it assumes some cultural similarities between Egypt and the broader Arab and Muslim world, this risks essentializing diverse and complex societies. Methodologically, conducting interviews online, though suitable and necessary in most cases, carries challenges common in digital research, including potential difficulties with rapport-building, loss of contextual cues, and occasional technical disruptions. In the future, building trust and rapport over a longer period of time to facilitate longer and perhaps in-person interviews would enrich the findings. Furthermore, because women were primarily recruited through online advertisements, the sample consisted mostly of middle-class and well-educated women, which constitutes a limitation of the study, as it may underrepresent the voices and experiences of women from lower socioeconomic classes, who might face additional barriers and forms of MSV. Therefore, the findings should be viewed as a starting point for further exploration in other parts and classes of the region. To expand the research, we plan to use this exploratory study to develop a questionnaire reflecting key manifestations of MSV for broader application across different contexts. Additionally, we aim to investigate the role of religiosity in shaping women's tolerance of such violence and examine the characteristics of both victims and perpetrators to better understand factors contributing to women's vulnerability.

## Data Availability

Anonymized demographic data is included in the body of the manuscript (Table 1). Further data that support the findings of this study are available on request from the corresponding author. The data is not publicly available due to containing information that could compromise the privacy of research participants.

## Appendix

### Interview Manual

1. Demographic characteristics (e.g., age, educational level, occupation, social class, duration of marriage, number of children).
2. How would you describe your sexual relationship with your husband?
3. Have there been times when you felt pressured to engage in sexual activity?
4. How did you feel during these instances?
5. How do you describe sex without your consent?
6. How do you define the term “marital rape"? Does it resonate with your experience?
7. How do religious teachings around marriage and sexual rights affect your perception of your experience?
8. How has your experience with marital sexual violence affected you emotionally, physically, and mentally?
9. How did it affect your relationship with your spouse or family? Has your husband ever had sex with you without your consent?
  Follow-up questions:
10. If you were to describe it in your own words, how would you describe or label the type of violence that you are subjected to?
11. Some people refer to this as what is known as marital rape. Would you describe it as marital rape?
  - If yes, why do you consider it rape from your point of view?
  - If no, why not, and how would you describe it instead?
